# Elevation of serum phosphorus, an early biomarker of acute kidney injury after cardiac sugery?

**DOI:** 10.1186/2197-425X-3-S1-A465

**Published:** 2015-10-01

**Authors:** J Ridolfo, M Saour, G Culas, N Zeroual, G Samarani, P Gaudard, P Colson

**Affiliations:** Arnaud de Villeneuve University Hospital, Department of Anesthesiology and Intensive Care, Montpellier, France

## Introduction

Acute kidney injury (AKI) is common after cardiac surgery and is a strong predictor of morbidity and mortality [1]. Hyperphosphatemia following AKI, by renal excretion defect, has never been studied in this context and could be a simple marker of AKI.

## Objectives

The aim of this study was to assess the predictability of serum phosphorus (Ph) for AKI monitoring after cardiac surgery.

## Methods

In this retrospective diagnostic validation study of 547 patients admitted in our institute between January 2012 and December 2012, we excluded patients with end stage renal disease (clearance < 15mL / min / 1.73m2) or dialyzed, solitary kidney or nephrectomy, lack of data. Serum creatinine (Cr) and Ph were measured preoperatively and postoperatively specifically (H0, H12, H24, H48, H72). The percentage of maximum elevation of Ph (%EPh = [(maximum -minimum) / minimum] * 100) was calculated. AKI was defined as an increase Cr more than 26.5 mmol / L in 48 hours according to KDIGO criteria [2].The diagnostic performance of postoperative Ph thresholds were analysed by elaborating area under the receiver operating characteristic curves (AUC-ROC) with sensitivity (Se), specificity (Sp), positive predictive value (PPV), negative predictive value (NPV).

## Results

From the 386 patients included, the mean Euroscore II was 4.2 ± 6.3%, SAPS II score, 26.4 ± 10.8. Among them, 21.2% developed AKI (grade 1: 13.2%, grade 2: 4.1%, grade 3: 3.1%) and 2.6% required renal replacement therapy (RRT). Patients with AKI had Euroscore II, duration of cardiopulmonary bypass, transfusion needing and mortality higher than those without AKI (p < 0.001). The %EPh and the Ph at 48 hours (Ph_48H_) were significantly higher in AKI patients than in no AKI patients: 81 ± 79% and 1.47 ± 0.46 mmol/l *vs* 25 ± 23% and 0.99 ± 0.2 mmol/L, respectively (p < 0.001). A value of Ph_48H_ > 1.19 mmol/L ( Se 72% (60-82), Sp 84% (71-92), PPV 84%, NPV 72%) and a %EPh > 49 % (Se 73% (61-81), Sp 83% (76-88), PPV 66%, NPV 86%) were predictive of AKI. In AKI patients, the %EPh and Ph_48H_ significantly increased with the severity of AKI (Table 1). In these patients, a Ph_48H_ < 1.53 mmol/L and a %EPh < 77% predicted the non use of RRT (Se 100% (62-100), Sp 85% (77-91), PPV 35% NPV 100%), respectively (Table 2).

**Table 1:** Table 1

AKI severity	%EPh	Ph_48H_ (mmol/L)
Grade 1	60 ± 45	1.25 ± 0.4
Grade 2	74 ± 58	1.73 ± 0.4
Grade 3	159 ± 132	1.80 ± 0.5

**Table 2:** Table 2

	Thresholds	AUC (IC95% )	*p value*
AKI diagnostic			
Ph_48H_	1.19 mmol/L	0.813 (0.735-0.890)	< 0.0001
%EPh	49%	0.830 (0.772-0.889)	< 0.0001
RRT requiring			
Ph_48H_	1.53 mmol/L	0.924 (0.879-0.970)	< 0.0001
%EPh	77%	0.818 (0.683-0.952)	< 0.0001

## Conclusions

After cardiac surgery, serum phosphorus seems to be a simple, reliable and inexpensive biomarker at bedside for AKI monitoring. A value less than 1.53 mmol/L at 48h may predict the no-initiation of RRT in case of AKI and may guide the clinician to a non-invasive-AKI therapeutic. Obviously, these results should be interpreted with caution regarding the retrospective nature of the study.

## References

[1] Lassnigg A, Schmidlin D, Mouhieddine M, Bachmann LM, Druml W, Bauer P (2004). Minimal changes of serum creatinine predict prognosis in patients after cardiothoracic surgery: a prospective cohort study. J Am Soc Nephrol.

[2] Kidney Disease: Improving Global Outcomes (KDIGO) Acute Kidney Injury Work Group. KDIGO Clinical Practice Guideline for Acute Kidney Injury. Kidney Inter. 2012, Suppl 2: 1-138.

